# Phytochemical profiling and anticancer potential of *gardenia latifolia* extracts against arsenic trioxide induced liver fibrosis in rat model

**DOI:** 10.3389/fphar.2024.1389024

**Published:** 2024-08-30

**Authors:** Zahid Mehboob, Sumaira Sharif, Madeeha Shahzad Lodhi, Abdul Bari Shah, Muhammad Romman, Iffat Nayila

**Affiliations:** ^1^ Institute of Molecular Biology and Biotechnology, The University of Lahore, Lahore, Pakistan; ^2^ Natural Products Research Institute, College of Pharmacy, Seoul National University, Seoul, Republic of Korea; ^3^ Department of Botany, University of Chitral, Chitral, Pakistan; ^4^ Department of Pharmacy, The University of Lahore, Sargodha Campus, Sargodha, Pakistan

**Keywords:** hepatocellular carcinoma (HCC), anticancer, cytotoxicity, histopathology, *G. latifolia*

## Abstract

**Introduction:**

Arsenic trioxide (As_2_O_3_) is an environmental contaminant that may cause hepatic injuries. As_2_O_3_-induced liver injuries are detected as an underlying cause of hepatocellular carcinoma (HCC) around the globe. The present study aimed to investigate the potential of *Gardenia latifolia* (GL) extracts against oxidative stress and apoptotic activity in HCC-induced rats and to explore *in silico* molecular docking analysis of phytocompounds of *G. latifolia*.

**Methods:**

The present study was designed to investigate the hepato-protective effect of ethanol and n-hexane extract of *G*. *latifolia*. Phytochemical analysis was performed using gas-chromatography-mass spectrometry (GC-MS), and the identified metabolites were used for computational docking analysis. The binding potential and inhibitory effect of the identified metabolites against inflammatory markers were assessed. Fifty male albino rats were selected for the *in vivo* study and were randomly divided into five groups, with 10 rats in each group. Group I is the control group. Hepatotoxicity was induced in groups II, III, IV, and V with 350 mg/kg/day of As_2_O_3_. Group II was taken as positive control, Group III and IV were treated with ethanol and n-hexane extract of *G*. *latifolia,* respectively, and Group V was treated with cisplatin 3.0 mg/kg/day. At the end of treatment, different stress and liver biomarkers were also analyzed.

**Results and Discussion:**

The quantitative phytochemical profiling revealed a high content of total flavonoid and tannins found at 5.731 ± 0.1856 mg quercetin equivalent (QE)/g and 86.31 ± 14.20 mg tannic acid equivalent (TAE)/g in *G. latifolia* n-hexane extract, while a significant concentration of TFC was 276.821 ± 2.19 mg gallic acid equivalent (GAE)/g, in ethanolic extract. GC-MS analysis resulted in the identification of 26 metabolites in ethanol extract while 32 metabolites in n-hexane extract, respectively. Both the extracts restored the abnormal levels of stress markers (*p* < 0.05) in Groups III and IV, and were comparable to the comparative control group V, which was given cisplatin as the standard drug. The histopathological examination revealed the regeneration of hepatocytes, dilated sinusoidal cells, necrosis, and distorted hepatic architecture observed in arsenic trioxide hepatotoxic liver. Among the top most identified metabolites from GC-MS analysis, stigmasterol exhibited −8.3 and −7.1 kcal/mol *in silico* binding affinities against cyclooxygenase-2 (COX-2), and interleukin (IL-6), respectively, while Dasycarpidan-1-methanol exhibited the best binding affinities of −6.8 and −7.2 kcal/mole against matrixmetalloprotinease (MMP)-3 and heat shock protein-90 (HSP-90), respectively. 6-AH-cAMP showed the best docking score of −7.5 kcal/mol for the vascular endothelial growth factor (VEGF) macromolecule. Metabolite Dasycarpidan-1-methanol, acetate represented drug like properties so it was further analyzed by MD simulation and stable dynamic nature of protein ligand complex was evaluated.

**Conclusion:**

In conclusion, the effective therapeutic potential of *G. latifolia* extracts targeted oxidative stress, increasing antioxidant activities and inhibiting inflammation and liver complications at early stages. Further research on the molecular level may further explore the anticancer potential of this plant against various types of cancers.

## Graphical Abstract

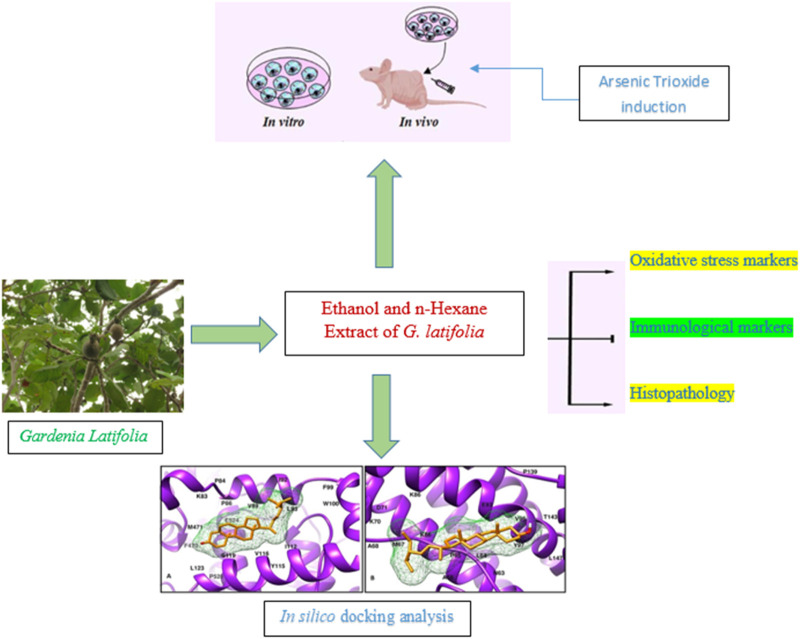

## 1 Introduction

Cancer is a deadly health condition characterized by the uncontrolled growth and spread of abnormal cells, leading to 9.6 million deaths, and ranking second among leading global killers in 2018 Lampertico et al., 2017. Hepatocellular carcinoma (HCC) is challenging to treat and ranks sixth among widely spread cancers globally. It is estimated that more than 90% of HCC cases develop in individuals with pre-existing liver diseases, and the greatest risk factor is cirrhosis (Lampertico et al., 2017; Marrero et al., 2018). In 2018, HCC was ranked 6th globally with 841,080 new cases, and over one million individuals are expected to be affected by this disease annually by 2025 (Shankar et al., 2019). The development of HCC is a complicated process involving sustained inflammatory injury that results in extracellular matrix (ECM) deposition, regeneration, and necrosis of hepatocytes (Pinyol et al., 2018). The most prevalent underlying causes of HCC development included viral infections, long-term alcohol consumption, fatty diet, aflatoxin, and environmental chemicals (Barsouk et al., 2021).

Arsenic (As) is widely present in water, soil, and rocks. According to the World Health Organization (WHO), higher levels (10 μg/L or more) of arsenic are reported in drinking water in many countries like China, India, Argentina, and the United States (Sander et al., 2015; Roy et al., 2021). This metalloid can enhance pathogen-associated molecular patterns (PAMPs) that may increase the inflammatory response, resulting in epigenetic and genotoxic effects. Also, these health hazards cause direct damage to tissues and organs by stimulating multiple signaling pathways, which may induce malignancies of the epidermis, lung, kidney, urinary bladder, prostate, and liver (Choiniere and Wang, 2016). Therefore, it is crucial to understand how arsenic intoxication may damage cells and to identify potential targets to treat its toxicity.

The liver is the principal organ for biochemical reactions and metabolism of toxins and xenobiotics, making it the main target of As_2_O_3_ (Abhilash et al., 2015). Arsenicals elicit hepatotoxicity by inducing the production of intracellular reactive oxygen species (ROS), which play a crucial role in the toxicity of arsenic and its derivatives by inducing cellular damage (Amin et al., 2012). Oxidative stress is a condition caused by an imbalance between antioxidation and oxidation (Nili-Ahmadabadi et al., 2018). Unbelievably, regardless of their etiology, all chronic hepatic disorders have a highly oxidative environment that perpetuates cellular damage and speeds up the development of fibrosis, cirrhosis, and, ultimately HCC (Conde-Hernández et al., 2017). Interleukin-6 (IL-6), interleukin-1β (IL-1β), and tumor necrosis factor-alpha (TNF-α) are key factors in initiating inflammatory responses by activating a series of systemic inflammatory cascades (Hu et al., 2021).

Natural substances are vital in cancer treatment, serving as adjuvants and chemotherapeutic agents. Phytochemicals, including leucovorin, Carzinophilin, vincristine, and actinomycin, have been crucial in cancer research as early anticancer drugs (Newman and Cragg, 2020). Taxus species, rich in taxanes, are essential in modern clinical practice as potent anticancer medications (Cragg and Pezzuto, 2016). However, they have a long history of being utilized in traditional medicine for the treatment of ovarian and breast cancers (Melo et al., 2018; Tewari et al., 2019). Turmeric and other curcuminoids inhibit NF-κB and Cyclooxygenase-2 (COX-2) activities, reducing prostaglandin formation (Desai et al., 2008).

*Gardenia latifolia,* a plant with medicinal properties, belongs to the Rubiaceae family and originates from East Asia. It has been extensively utilized as both food and medicine in South Asian countries, primarily for treating digestive issues (Alshabi and Shaikh, 2022). There are other reports of its use to treat and cure colic, stomach aches, and sexual illnesses (Amidi et al., 2012; Sundar and Habibur, 2018; Saravanakumar et al., 2021). Research has demonstrated the anticancer and antioxidant properties of *G. latifolia* leaves and barks (Reddy et al., 2021).

Therefore, the current study is presented to demonstrate the therapeutic efficacy of *G latifolia* ethanol and n-hexane extracts for their potential against As_2_O_3_-induced liver cancer by investigating *in vitro*, *in vivo*, and *in silico* efficacy.

## 2 Materials and methods

### 2.1 Chemical and reagents

Analytical-grade chemicals and solvents were used in this study. Ethanol and n-hexane were obtained from Merck (Sigma, Texas, United States, lot no. 64-17-5). Sodium carbonate solution (7%), NaNO2 (5%), AlCl3 (10%), DPPH, Ascorbic acid, Hydrogen peroxide (20 mM), PBS, Sodium nitroprusside, Nitroblue tetrazolium, MTT, Cisplatin, DMSO, Ketamine/xylazine, Normal saline, Formalin (5%), MDA, EDTA, TCA (5%), Potassium iodide, Hematoxylin, Eosin were used in appropriate tests as described in methodology of this research work.

### 2.2 Plant collection and extraction

*Gardenia latifolia* (Papara) was collected from the local market of Lahore, Pakistan. The plant was identified by a taxonomist/Professor at the Department of Botany, Government College University Lahore. The plant parts (leaves, stems and shoots) were oven-dried at 40°C for 3 days, grounded, and stored in an airtight container. A total of 400 g of the powdered plant was extracted with 1,000 mL of 70% ethanol and n-hexane solvents, thoroughly agitated, and stored at 25°C for 2 weeks. Afterward, the extract was filtered with Whatman no.1 filter paper. The plant extracts were concentrated using a lyophilizer (Labconco Corporation, Shanghai, China, lot number 140616) and stored at 4°C for further analysis.

### 2.3 Estimation of phytochemicals

#### 2.3.1 Total phenol content (TPC)

Folin-Ciocalteu reagent 1 mL was mixed with each extract (1 mL of ethanolic extract and 1 mL of n-hexane extract), and sodium carbonate solution (7%) 10 mL was added to the mixture, followed by vigorous mixing and incubated at room temperature (RT) for 90 min before the absorbance was taken at 760 nm by using Benchmark spectrophotometer. The standard curve was generated using Gallic acid as standard in water (10–50 mg/L). TPC was measured and expressed in mg Gallic acid equivalent (GAE) per Gram dry sample extract.

#### 2.3.2 Total flavonoid content (TFC)

Ethanol extract (1 mL) and n-hexane extract (1.4 mL) and a standard quercetin solution were mixed with 4 mL of dH2O, followed by 0.3 mL of NaNO_2_ (5%). Then, 0.3 mL of AlCl_3_ (10%) was added after 5 min, and the resulting solution was mixed with 2 mL of NaOH (1 M) and diluted with 2 mL of dH2O. Then, absorbance was measured at 510 nm against blank. The results were estimated as mg equivalent of Quercetin equivalent (QE) per Gram of dried plant sample.

#### 2.3.3 Total tannin content (TTC)

To, 0.5 mL of each extract (ethanolic extract and n-hexane extract) of *G. latifolia*, mixed with 20 mM ferric chloride and 8 mM of potassium ferric cyanide prepared in hydrochloric acid (0.1 M). The contents were combined, and the absorbance was taken at 700 nm against blank. Tannic acid was served as a standard reagent. The results were calculated as mg Tannic acid equivalent (TAE)/g of dried plant sample.

### 2.4 Evaluation of antioxidant activities

#### 2.4.1 DPPH radical scavenging assay

The DPPH radical quenching capacity of all plant extracts was estimated by a previously described method with slight modification (Blois, 1958). Following the preparation of a 0.1 mM DPPH solution in methanol, 1 mL of this solution was added to 3 mL of methanol containing different concentrations (10–50 μg/mL) of each plant extract. After thirty minutes of incubation in the dark, the absorbance was measured at 517 nm using a spectrophotometer. Ascorbic acid was used as a reference reagent. Each experiment was conducted in triplicate. The calculation for the scavenging activity was estimated by the following formula:

DPPH Free radical scavenging %=A control−A plant extracts/A control×100

#### 2.4.2 H_2_O_2_ scavenging assay

The assay utilized to ascertain the hydrogen peroxide scavenging capacity of the plant extract was given previously by (Jayaprakasha et al., 2004). A 20 mM solution of hydrogen peroxide was formulated in phosphate buffer saline [PBS; pH 7.4]. 1 mL of each sample having various concentrations (10–50 μg/mL) of each extract was added into 2 mL of freshly prepared hydrogen peroxide solution. All absorbances were measured at 230 nm after 10 min, in comparison to a blank solution comprising hydrogen peroxide solution devoid of the extract. The calculation for the percentage of H_2_O_2_ radical scavenging was done by the following formula:

H2O2 radical scavenging %=A control−A plant extracts/A control×100

#### 2.4.3 Nitric oxide scavenging assay

The nitric oxide quenching ability of selected plants was determined by a previously reported method described by (Garratt, 2012). A 2 mL solution of sodium nitroprusside (10 mM; pH 7.4) was combined with 0.5 mL of each extract and ascorbic acid at concentrations ranging from 10 to 50 μg/mL. Following 150 min, 0.5 mL of Griess reagent [1.0 mL of sulfanilic acid reagent (0.33%)], 0.5 mL of naphthyl ethylene diamine dihydrochloride (0.1% w/v), and 20% glacial acetic acid was added in 0.5 mL of incubation solution. After 30 min of incubation at room temperature, the absorbance was taken at 540 nm using a spectrophotometer.

$$\text{NO radical scavenging }\left(\%\right)=\left(A\text{ control}-A\text{ plant extracts}\right)/\text{A control}\times 100$$

#### 2.4.4 Super oxide scavenging assay

The antioxidant activity of all extracts was evaluated using the previously documented methods with slight changes (Nishikimi et al., 1972). A reaction mixture containing I mL of nitro blue tetrazoliumn (156 μM NBT in 100 mM PBS; pH 7.4), 1 mL of NADH solution (468 μM in 100 mM phosphate buffer; pH 7.4), and 1 mL of plant extracts (10–50 μg/mL) was added. A 100 μL volume of phenazine methosulphate (PMS) solution (60 μM PMS in 100 mM phosphate buffer; pH 7.4) was introduced into the mixture to initiate the reaction. After 5 min of incubation, the absorbance was taken at 560 nm against a control sample. The inhibition of free radicals was calculated using the following formula:

$$\text{Superoxide anion radical scavenging }\left(\%\right)=\left(A\;control-A\;plant\;extracts\right)/A\;control\times 100$$

### 2.5 GC-MS-analysis

The GC-MS analysis of the ethanol and n-hexane extracts was carried out using Agilent GC-MS triple quad 7000 A (GC-MS- 7890 A) (Hitechi instruments, Tokyo, Japan) interfaced with EI and CI ion sources. The experimental condition of the GC-MS system contained DB 35 MS capillary standard non-polar column, dimension 30 m, ID: 0.25 µm film thickness. Helium was set as a carrier gas at 1 mL/min. The initial temperature profile was 150°C (hold time 5 min), and finally, the temperature was increased to 310°C. The injection volume was 50 µL. The results were compared using the National Institute of Standards and Technology (NIST) library program. The NIST library provides information regarding compound names, molecular weight, and chemical formulas. The relative percentage of each metabolites was determined using a peak area from the chromatogram, which is automatically done by calculations.

### 2.6 *In Vitro* evaluation of anticancer activity

#### 2.6.1 Culturing of cell lines

Human Liver Cancer (HepG2) cell lines were maintained in RPMI 1640 medium buffered with 2 g/L of HEPES and sodium bicarbonate, and in a humidified atmosphere containing 5% CO_2_ at 37°C. Both the cell lines were grown in media (DMEM-HG) augmented with FBS (10%), streptomycin, and penicillin in a culture flask. The culture was administered to the developing cells at 70%–80% confluency. Following a 1xPBS rinse and trypsin-EDTA treatment to the point when the adherent cells were expelled from the flask’s surface, the flask’s cell separation was verified with an inverted microscope (Arodin et al., 2019).

#### 2.6.2 Measurement of cells viability

The *G. latifolia* ethanol and n-hexane extracts were used to calculate the IC_50_ value and the percentage of cell viability using the 3-(4,5-dimethylthiazol-2yl)-2,5-diphenyl-2H-tetrazolium bromide (MTT) assay and compared with the effect of the standard drug cisplatin (Kalsoom et al., 2022). The cells were seeded in 96 well plates and left to culture for 24 h. The cells were then treated with cisplatin and *G. latifolia* extracts at various concentrations (10, 20, 50, 100, and 200 μg/mL). Then, the cells were cultured again for 72 h in an incubator. Following 24 h of exposure to the extracts, 100 µL MTT solution was added, incubated, and promptly removed. The monolayer cells were gently rinsed twice with PBS (Invitrogen Inc., Texas, United States, lot no. 524650). To each well, 100 µL of dimethyl sulfoxide (DMSO) was added for dissolving the formazan crystal. The optical density (OD) of the samples at 570 nm was monitored using a BIOBASE microplate reader (BioTek-ELx800) (Shandong, China).

### 2.7 *In vivo* studies

A total of 50 male albino rats, weighing between 100 and 120 g with an average age of 6–7 weeks, were taken from the IMBB, UOL animal house. The rats were given a regular pellet meal from Pet Store Lahore, Pakistan, Brit rat food, and water. They were retained in a light and dark cycle for 12 hours at a temperature of 25°C. The study was approved (Ref Number IMBB/CRiMM/21/1020) by the ethical and review board committee of the IMBB.

#### 2.7.1 Experimental design

A total of fifty albino rats were randomly divided into five groups, each group containing 10 rats. Group I was the control group and was given only normal saline (0.9%). Groups II, III, IV, and V were intraperitoneally administered with arsenic trioxide (As_2_O_3_) at the dose of 350 mg/kg/day for 28 consecutive days. After a week the treatment was initiated in all experimental groups as; Group II was considered positive control and given no treatment. Groups III and IV were treatment groups, administrated with *G*. *latifolia* ethanol and n-hexane extracts by intra-gastric route at a dose of 200 mg/kg/day, respectively, Group V was a comparative control group, administered with Cisplatin 3.0 mg/kg intraperitoneally. The rats of each group were sacrificed at the termination of the treatment period (after 4 weeks) using intraperitoneal ketamine/Xylazine injection with a dose of 50 mg/kg ketamine and 10 mg/kg xylazine (Ketamine lot no. 3511021, 50 mg/mL; Arevipharma GmbH, Radebeul, Germany). After cardiac puncture, blood serum was obtained from whole blood by leaving it to coagulate for 20 min at 4°C, then centrifuged at 4,000 rpm for 15 min. The liver, lobes, and kidney tissues were separated after dissection and were preserved in 5% formalin for histopathological testing (Irshad et al., 2021).

### 2.8 Estimation of oxidative stress markers

#### 2.8.1 Estimation of malondialdehyde (MDA)

A reactive material to thiobarbituric acid was employed to calculate the serum MDA level Byambaragchaa et al., 2014. A 10% (w/v) homogenate was made from 1 mL of serum using a 10 mM buffer (pH 7.4). For immediate thiobarbituric acid reactive compounds, the supernatant was employed. The following ingredients were used in the test: blood sample, sodium dodecyl sulphate (8.1%), TBA (1.5 mL, 0.8%), acetic acid (1.5 mL, 20%) solution (pH 3.5), distilled water (4.0 mL), and n-butanol (5.0 mL). The absorbance of the homogenate was then taken at 532 nm with the help of a spectrophotometer (Benchmark BMS430) (X-light, Michigan, United States of America).

#### 2.8.2 Estimation of glutathione (GSH)

The technique developed by Irshad et al., 2021 was used to measure glutathione reductase. It helps turn oxidized glutathione into reduced glutathione. NADPH concentration serves as a direct indicator of the subsequent enzyme activity since glutathione reductase uses NADPH to catalyze the conversion of oxidized glutathione to reduced glutathione. Oxidized glutathione (0.1 mL) and the sample (0.1 mL) were dispensed in a cuvette with the addition of 600 µL of distilled water gain volume up to the final 2 mL. The mixtures were incubated with NADPH (0.1 mL) for 3 min. *A*bsorbance at 340 nm was measured every 15 s for two to 3 minutes. Water was used as a control instead of oxidized glutathione. The GSH activity was estimated in nanomoles of NADPH oxidized/minute/g of material.

#### 2.8.3 Estimation of glutathione peroxidase (GPx)

The serum sample (0.1 m*L*) was first homogenized with 2.4 m*L* of EDTA (0.02 M) *and* w*as* kept on ice for 10 min. *D*istilled water (3 m*L*) was mixed with 0.5 m*L* of 50% TCA, and placed on ice for 10 min. The mixture was subjected to centrifugation for 10 min at 3,000–3,500 rpm. A test tube containing 1 m*L* of the supernatant and 2 m*L* of Tris HCL (0.15 M) and DTNB (0.05 mL) w*as* used to measure the absorbance at 412 nm (Ben-Abdallah et al., 2021).

#### 2.8.4 Estimation of nitric oxide (NO)

NO undergoes *a* reaction with oxygen to produce nitrite ions, the amount of which may be calculated using the Greiss reagent. NO scavengers contend with oxygen, resulting in less nitrite ion formation. The experiment involved mixing various doses of n-hexane and methanol extracts of the plants under study with 10 mM sodium nitroprusside in phosphate saline buffer, incubated at RT for 2.5 h. The control was the same *as the* mixture *of* n-hexane and ethanol without *plant* extract*s* (Gil et al., 2021).

#### 2.8.5 Estimation of catalase activity (CAT)

A 10% (w/v) homogenate prepared from serum (1 mL) was centrifuged at 3,000 rpm for 10 min at 4°C using a 10 mM buffer (pH of 7.4). The resultant supernatant was employed for which phosphate buffer (50 mM), 100 µL of supernatant, and freshly made H_2_O_2_ (30 mM). At 240 nm, the rate of H_2_O_2_ oxidation was determined spectrophotometrically. CAT activity was measured in µmol/mL of protein.

#### 2.8.6 Estimation of advanced oxidative protein products (AOPPs)

A 200 µL of blood plasma was dispensed in a microtiter plate (Becton D Labware, Lincoln Park, New Jersey, United States (lot no 238901). The plasma was diluted with PBS (1/5) acetic acid (20 mL). The reaction mixture’s absorbance was immediately measured on the microplate reader at 340 nm in comparison to a blank that contains PBS (200 mL), potassium iodide (10 mL), and acetic acid (20 mL).

#### 2.8.7 Estimation of superoxide dismutase

Blood serum was extracted, homogenized in 50% TCA, and spun at 13,000 rpm. The SOD activity was assessed using the supernatant. The following ingredients were used in this test: 100 µL serum sample with SPB, 1.2 mL (pH 8.3, 0.052 M), 100 µL phenazine methosulphate with a concentration of 186 M, 200 µL of NADH (750 M), 300 µL nitro blue tetrazolium (300 M), and n-butanol (4.0 mL). NADH was dispensed to start the reaction. Against butanol, its absorbance at 560 nm was measured.

#### 2.8.8 Estimation of liver function tests

The activities of aspartate aminotransferase (AST), alanine aminotransferase (ALT), alkaline phosphatase (ALP), albumin, and globulin were evaluated in the serum of experimental rats. These were carried out using ELISA Kits lot No. 199062 (Sigma Aldrich, Massachusetts, United States). The protocols were used as stated in the manufacturer’s manuals. AST and ALT activities in serum were measured with a spectrophotometric method, whereas the colorimetric determination of ALP, albumin, and globulin activity was carried out using commercial kits.

### 2.9 Histopathology

An automated Kalstein tissue processor having Lot No.115610 (Sakura, Japan) dehydrated, clarified and impregnated the fixed liver tissues overnight. Serial sections of four thicknesses were cut from the specimens by a microtome. The sections were then embedded in paraffin blocks via an embedding machine (Model RM2245, Leica Biosystems, Halle, Germany). Hematoxylin-eosin was used to stain the sections following accepted pathologic practices (Chaudhry et al., 2021). A series of washes for 1 minute in 70, 80, 96, and 100% ethanol was used to rehydrate sections, cleaned with distilled water, HCL (0.1%) in ethanol (50%), tap water for 10 min, hematoxylin for 2 min, eosin for 1 min, and distilled water. The slides were mounted with coverslips after being dehydrated with 95%, 100%, and then xylene for 25 min. Hematoxylin-eosin-stained slices of the anomalies were examined using an epifluorescence microscope (DM LB2, Leica, Wetzlar, Germany) to compare structural changes. A CCD digital camera was used to capture images of H-E staining at original magnifications of 40.

### 2.10 Immunological biomarkers

Following Immunological biomarkers were estimated in all experimental groups using commercially available ELISA kits (Abcam, Cambridge, MA, United States) based on sandwich ELISA protocol, COX-2, matrix metalloproteinase 3 (MMP-3), HSP-90, VEGF, and IL-6. ELISA assay was used to ascertain and measure biomarkers in the sample. Antibodies coated on the plate bind to the target protein/antigen to identify the presence of the protein to be targeted. All the reagents of the kit were brought to room temperature (20°C–25°C) before use. All reagents were prepared according to the instructions mentioned in kit protocols and the amount of bounded protein was estimated through a standard curve (Gülçin et al., 2004).

### 2.11 Molecular docking studies

Molecular docking was used to map out how isolated bioactive metabolite from plant extracts in ethanol and n-hexane interacted with the proteins involved in cancer pathways. This study used PyRx tools AutoDock Vina to proceed with the molecular docking analysis. The PyRx 1.0 virtual screening docking software program was downloaded, installed, and used to perform molecular docking studies of identified metabolites. ChemSpider was used to retrieve the three top docked phytocompounds (from GC/MS analysis data of plant extracts) against selected target cancer proteins COX-2, IL-6, Vascular endothelial growth factor (VEGF), matrix Metallopeptidase 9 (MMP-9) and Heat shock protein-90 (HSP-90).

### 2.12 Statistical analysis

The data obtained from all experiments was employed as mean ± standard deviation (SD). One-way ANOVA Tukey’s multiple comparison tests were used to compare groups at the significance level *p* < 0.05, *p* < 0.001, and *p* < 0.0001. For the analysis, the whole data Graph pad Prism, 9.1.2 software (Prism Academy, Boston, United States) was employed.

## 3 Results

### 3.1 Total phenolics flavonoids, and tannin contents

**FIGURE 1:**
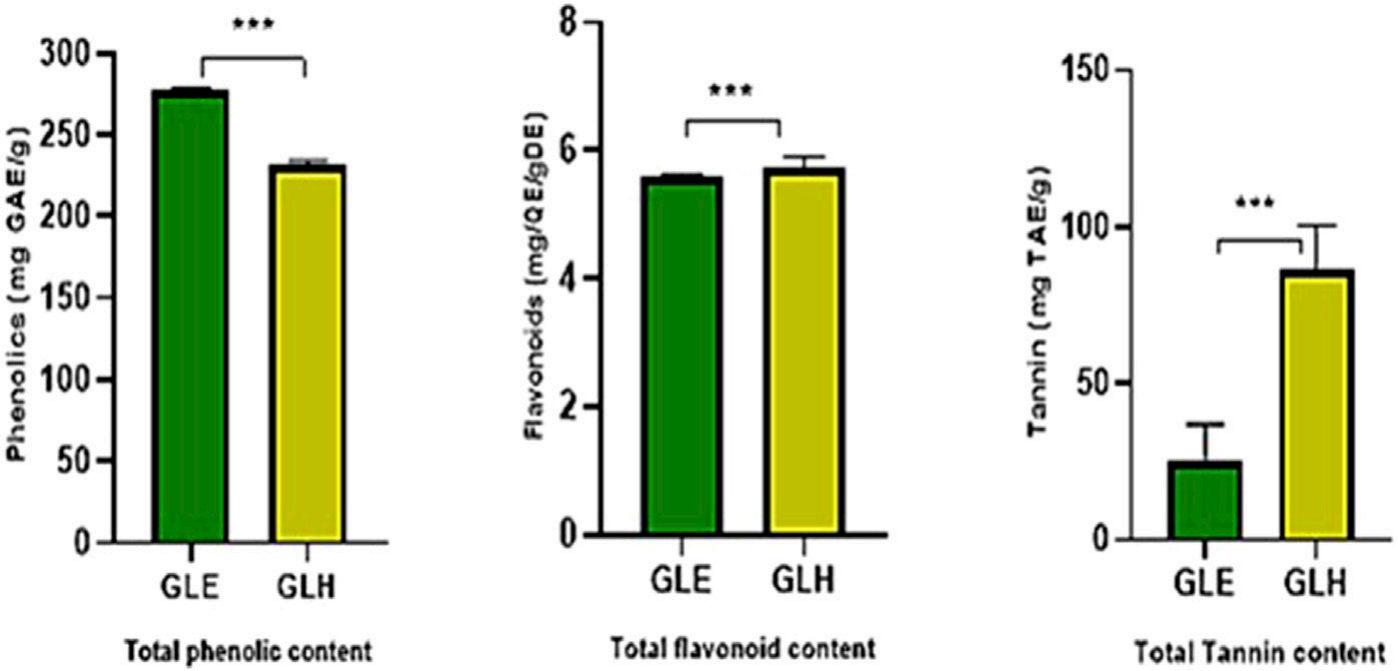
Total phenolics, flavonoids, and tannins in ethanolic and n-hexane extracts of *Gardenia latifolia*. Data are expressed as mean ± SD and statistical significance is considered as *** (*p* < 0.0001). Abbreviations: GAE, Gallic acid equivalent; QE, Quercetin equivalent; TAE, Tannic acid equivalent; GLE, ethanolic extract of *Gardenia latifolia*; GLH, n-hexane extract of *Gardenia latifolia.*

The phytochemical estimation resulted in higher values of the metabolites. The mean values of TPC obtained from ethanol and n-hexane extract were 276.821 ± 2.19 and 231.643 ± 2.24 mg GAE/g of the dried extract, respectively. TFC was estimated for ethanolic and hexane extracts to be 5.583 ± 0.05774 and 5.731 ± 0.1856 mg QE/g of the dry extract. The TTC in ethanol and n-hexane extracts of *G. latifolia* were found to be 25.04 ± 11.9 and 86.31 ± 14.2 mg TAE/g of dry extract, as presented in Figure 1. The highest amount of phenolics metabolite was found in *G*. *latifolia* ethanol extract, and higher tannin concentrations were found in n-hexane extract.

### 3.2 Evaluation of the antioxidant activities

**FIGURE 2:**
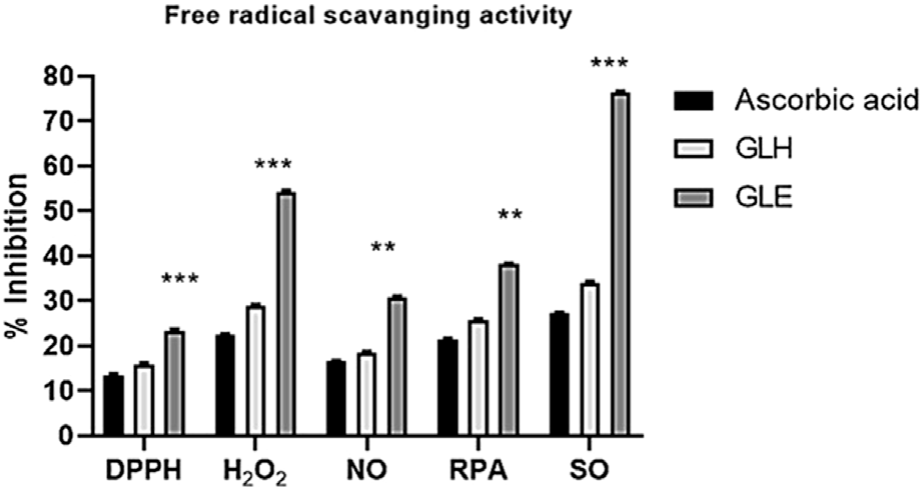
Antioxidant activities of ethanolic and hexane extracts of *Gardenia latifolia* against various oxidants. Data are expressed as mean ± SD and statistical significance is considered as *p* < 0.001 and *p* < 0.0001. Symbols indicated significant levels as **(0.001) and ***(0.0001). DPPH, 2,2-Diphenyl-1-picrylhydrazyl; H_2_O_2_, Hydrogen peroxide; NO, Nitric oxide; RPA, reducing power assay; SO, Superoxide anion; GLE, ethanolic extract of *Gardenia latifolia*; GLH, n-hexane extract of *Gardenia latifolia*.

Different assays were performed to estimate the antioxidant potential of the ethanolic and n-hexane extracts. In all oxidant scavenging assays, as shown in Figure 2, the highest antioxidant activity was by the *G. latifolia* ethanol extract compared to ascorbic acid and n-hexane extract. DPPH and NO scavenging activities of ethanol extract were observed to be higher as associated to standard. These results were compared with ascorbic acid used as a standard reagent. The extract showed substantial antioxidant activity similar to ascorbic acid, which was used as a control standard antioxidant. A considerable inhibition of the DPPH radical activity was observed when ethanolic extract of the plant was used but pharmacological effectiveness is still needed to be assessed to indicate therapeutic efficacy of theses extracts. The ethanolic extract also showed good NO scavenging activity, reducing the nitrite concentration in the assay medium. Collectively, these results suggest that *G. latifolia* ethanolic extract effectively scavenges RPA and SO.

### 3.3 GC-MS analysis

GC-MS analysis of the ethanolic extract of *G. latifolia* resulted in the identification of a total of 27 metabolites belonging to various groups of organic compounds. A list of the metabolites along with their names, molecular formula, molecular weight, retention time (RT), and class of compounds are presented in Supplementary Table S1. Similarly, the GC-MS analysis of n-hexane extract revealed the identification of a total of 32 metabolites from various classes of organic compounds. The list of the compounds identified in n-hexane extract is given in Supplementary Table S2.

### 3.4 *In vitro* evaluation of anticancer activity

#### 3.4.1 MTT assay

**FIGURE 3:**
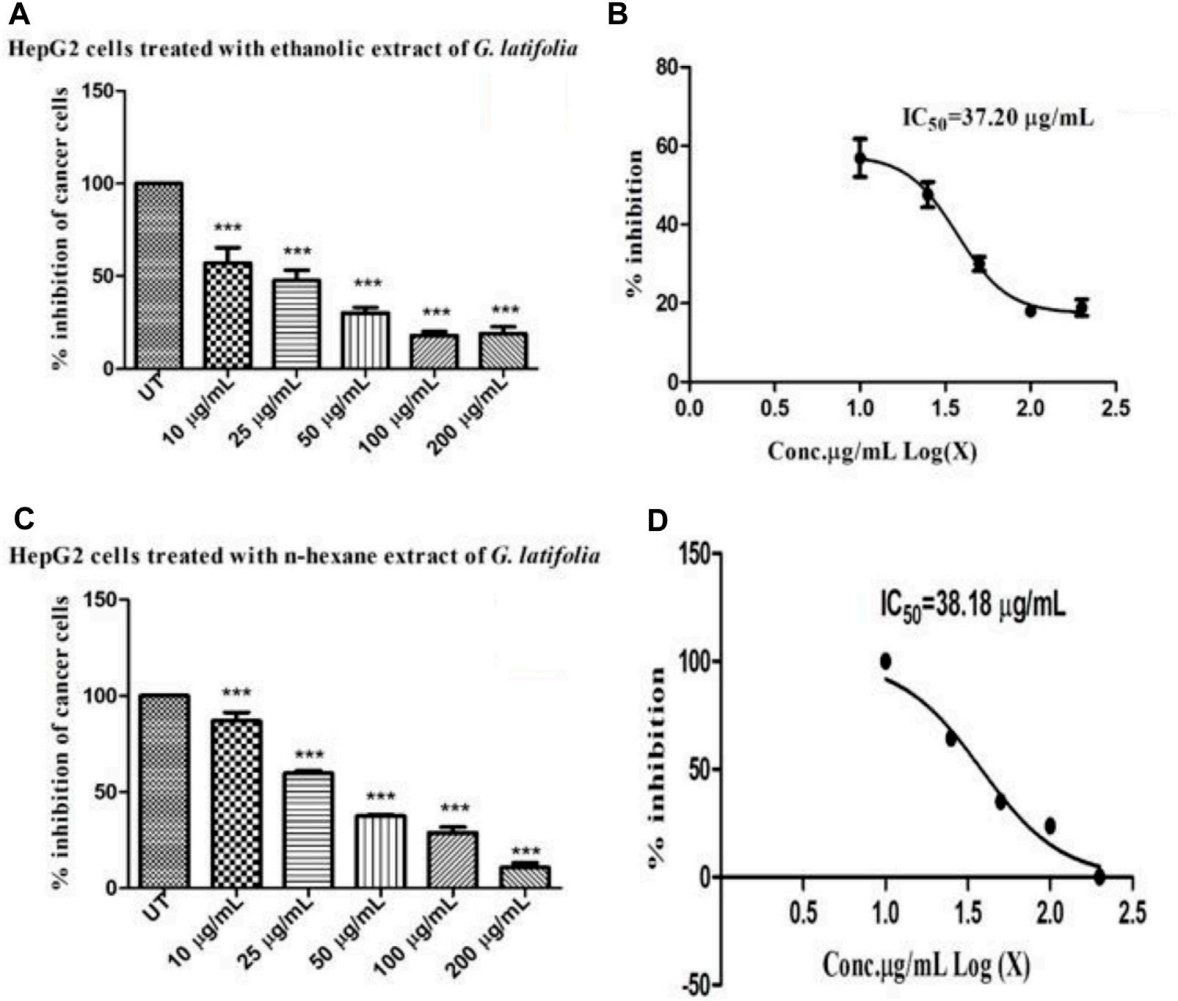
Cytotoxic effect of ethanolic and n-hexane extract of *Gardenia latifolia* determined by MTT assay. **(A)** % of inhibition of HepG2 cells following treatment with ethanolic extract of *Gardenia latifolia* **(B)** IC_50_ concentration estimated for ethanolic extract of *Gardenia latifolia* **(C)** % of inhibition of HepG2 cells following treatment with n-hexane extract of *Gardenia latifolia and* **(D)** IC_50_ concentration estimated for n-hexane extract of *Gardenia latifolia.* UT (untreated). Data are expressed as mean ± SD and statistical significance is considered as *p* < 0.0001 and the symbol indicates a significant level *** (0.0001) in comparison to the UT-HG untreated group. MTT, 3-(4, 5-dimethylthiazol-2yl)-2,5-diphenyl-2H-tetrazolium bromide.

The MTT assay was performed triplicate to determine the cytotoxicity of the ethanolic and n-hexane extracts of *G. latifolia*. The results are presented in Figure 3 which demonstrates cytotoxicity as the %age inhibition of cells corresponds to the concentrations (10, 50, 100, and 200 μg/mL). At higher concentrations, both the ethanolic and n-hexane extracts exhibited concentration-dependent toxicity in the HepG2 cells. The IC_50_ values for *G. latifolia*, extracts in ethanol (Figure 3B) and n-hexane (Figure 3D) were observed to be 37.20 and 38.18 μg/mL, respectively. The findings showed that GLE extract possesses higher cytotoxicity (lower IC_50_ value) than GLH extract. A significant difference was found in the toxicity level of ethanol and n-hexane extracts of the plant under study.

### 3.5 *In vivo* studies

**FIGURE 4:**
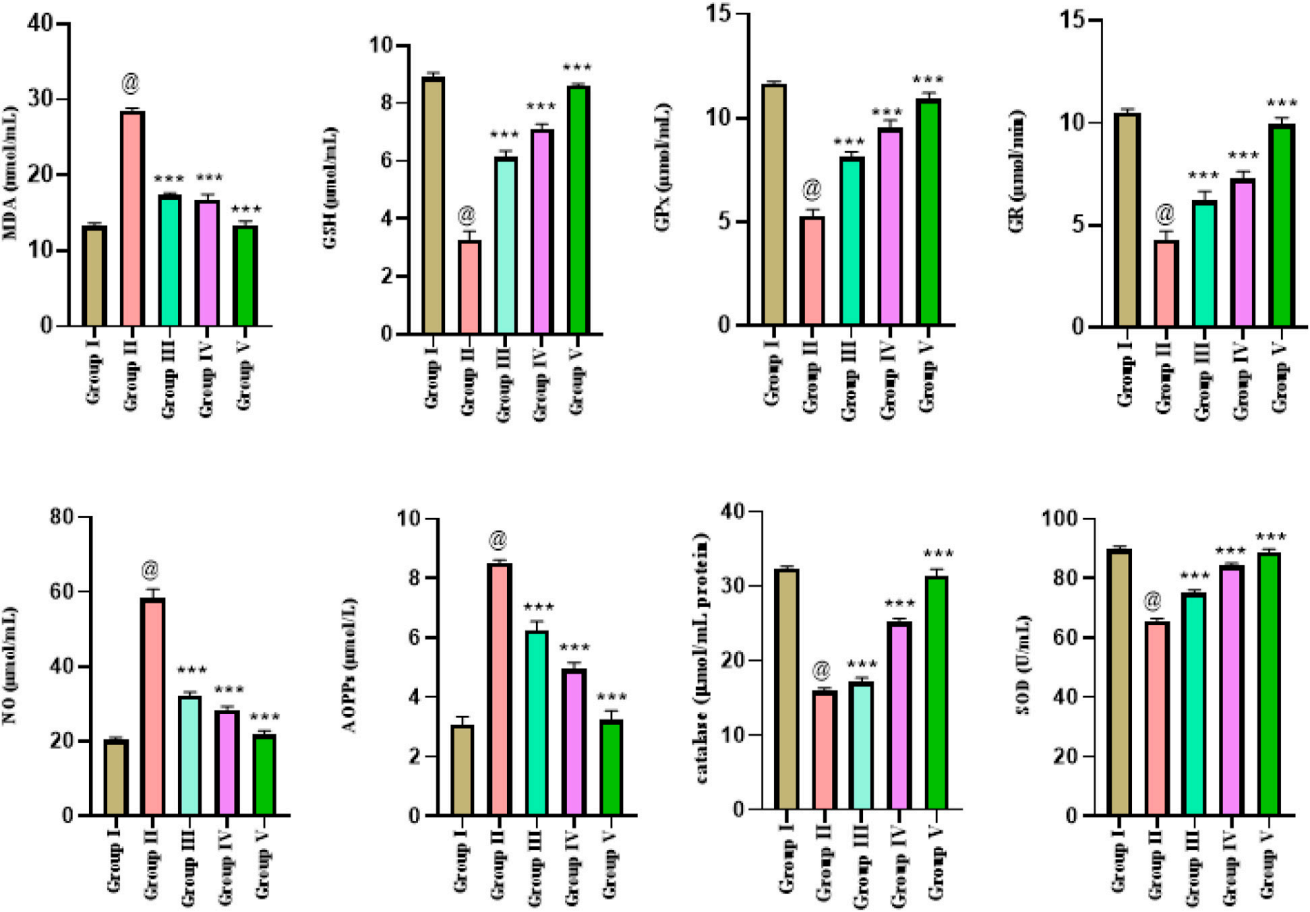
Estimation of oxidative stress parameters in liver cancer-treated animals with ethanol and n-hexane extracts of *Gardenia latifolia*. Here, Group I; Control group, Group II; Arsenic treated group, Group III; ethanol extract treated group, Group IV; n-hexane treated group, and Group V; Cisplatin-treated group. Data are expressed as mean ± SD and statistical significance is considered as *p* < 0.0001. The symbol @ indicated a significant level of the diseased reference group, while *** (0.0001) indicated a significant level of treatment groups compared to the diseased reference group (group II). SOD, superoxide dismutase; MDA, malondialdehyde; NO, nitric oxide; GSH, glutathione; GPx, glutathione peroxidase; GR, glutathione reductase; AOPPs, advanced oxidative protein products.

The oxidative stress results in rats (n = 50) in five different experimental groups were presented as mean ± SD and analysis was performed triplicate. Analysis of the comparison of means values of all parameters was employed through one-way ANOVA. Significantly higher mean levels of MDA 28.52 ± 0.009 were observed in positive control group II, and it was restored in treatment groups III and IV with ethanol extract and n-hexane extract. The lower GSH levels were reinstated (close to the control group) in all the treated groups as 6.131 ± 0.022 in group III and 7.131 ± 0.015 in group IV. A significant statistical difference in mean glutathione values was observed in all the treated groups compared to group II. Significantly lower mean GPx value of 5.276 ± 0.032 was observed in group II. A significant statistical difference in mean GPx levels was observed in all the treated groups compared to positive control group II. The mean GR level 4.298 ± 0.041 decreased significantly in positive control group II. However, the restoration of normal levels of GR were higher in all treated groups. Similarly, NO restoration levels were high in all the treated groups. Significantly higher mean levels of AOPPs 8.523 ± 0.009 were observed in group II. The normal CAT levels (close to the control group values) were reinstated in treated groups as 17.17 ± 0.054 in group III, 25.17 ± 0.052 in group IV, and 31.39 ± 0.917 in group V. The mean value of SOD in group I was recorded as 89.75 ± 0.024, and the mean SOD level (65.6 ± 0.1708) decreased significantly in group II. The lower SOD levels were reinstated in all the treated groups as 75.13 ± 0.039 in group III and 84.13 ± 0.025 in group IV 88.85 ± 0.539 in group V rats (Figure 4).

### 3.6 Liver function tests

**FIGURE 5:**
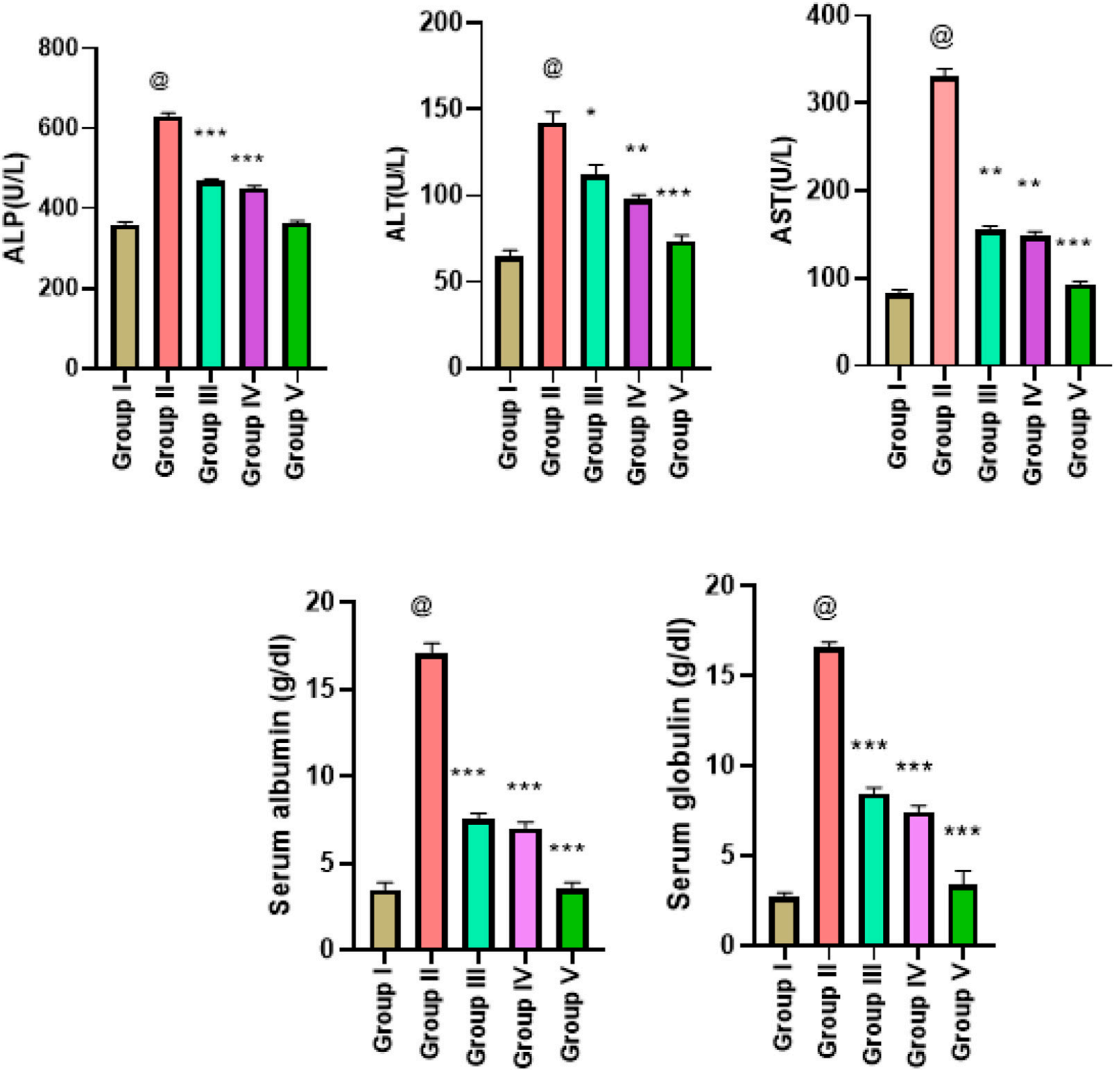
Estimation of liver function tests in liver cancer-treated animals with ethanol and n-hexane extracts of *Gardenia latifolia.* Here, Group I; Control group, Group II; Arsenic treated group, Group III; ethanol extract treated group, Group IV; n-hexane treated group, and Group V; Cisplatin-treated group. Data are expressed as mean ± SD, with statistical significance *p* < 0.05, 0.001, and *p* < 0.00010. The symbol @ indicated a significant level of the diseased reference group while *(0.05), ** (0.001), and ***(0.0001) indicated a significant level of treatment groups in comparison to the diseased reference group (group II). AST, aspartate aminotransferase; ALT, alanine aminotransferase; ALP, alkaline phosphatase.

The results of LFTs in five different experimental groups are presented as mean ± SD. Analysis was performed triplicate and comparison of means values of the parameters was employed by a 0.05 significant level (*p*-value) obtained through one-way ANOVA. Significantly higher levels of ALP were observed in group II. The higher ALP levels were restored in treatment groups III and IV with *p* < 0.05. The ALT level in group I was increased significantly (*p* = 0.0001) and in positive control group II. The normal ALT levels (close to the control group values) were reinstated in treated group III (*p* < 0.05) and in group IV (*p* < 0.001). The mean value of AST in group III was significantly improved (*p* < 0.0010 with *G. latifolia* ethanolic extract treatment. Higher albumin level in group II was decreased significantly (*p* = 0.0001) in groups III and IV with ethanolic and hexane extract-treated rats. The lower globulin levels were reinstated in ethanol-treated and hexane extract-treated group IV with a significant *p*-value of 0.0001. All LFT values are depicted in Figure 5.

### 3.7 Histopathological analysis

**FIGURE 6:**
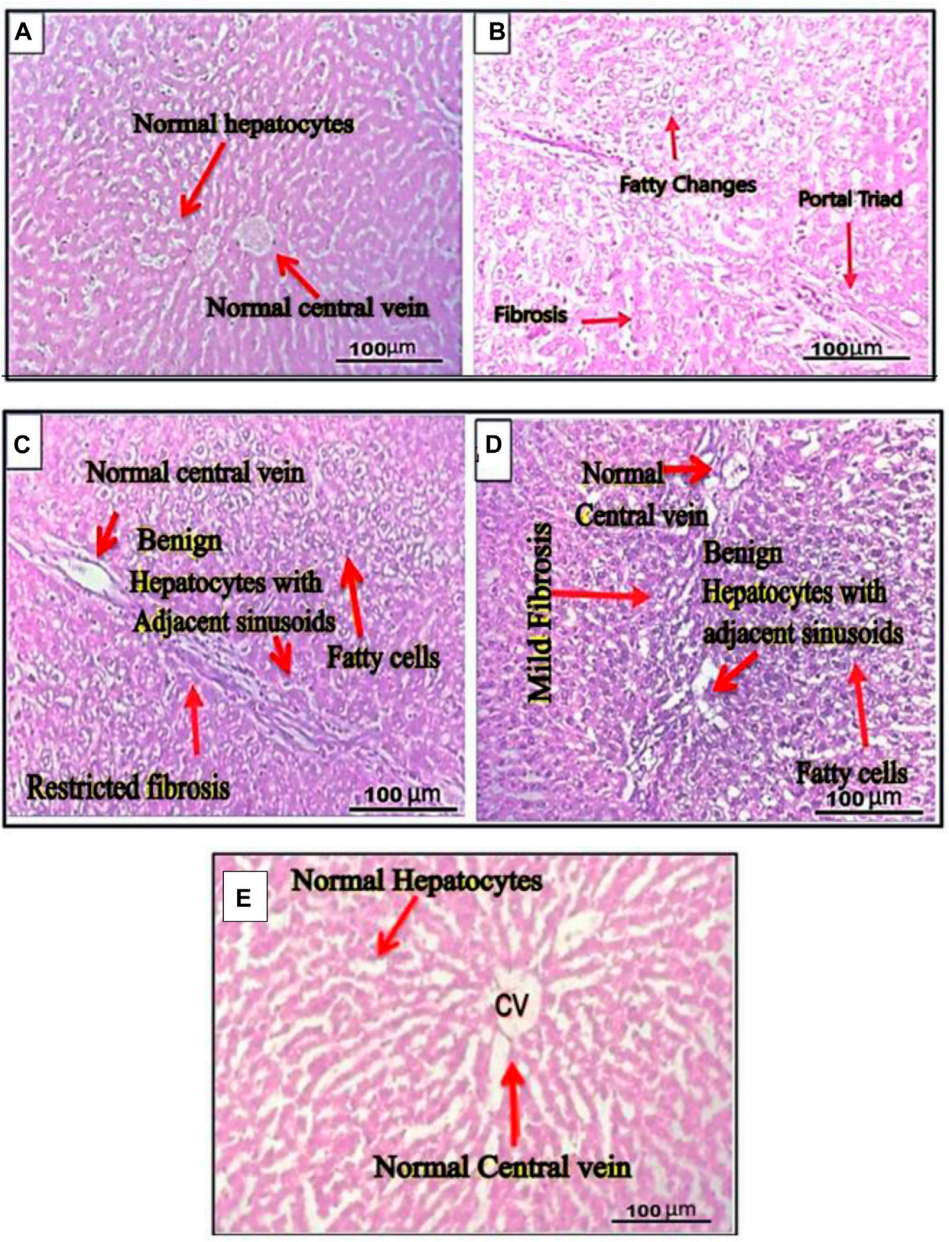
Cross sections of rat liver. **(A)** The section of the normal liver revealed parenchyma with rows of benign hepatocytes and adjacent sinusoids. The portal triad and central vein were also unremarkable. **(B)** The liver section of rats belonging to group II treated with arsenic trioxide showed hepatic fatty changes, sinusoidal congestion, central hepatic venous congestion, and severe fibrosis. **(C)** Cross sections of rat liver from groups III administered with ethanol extract. **(D)** The liver section of group IV rats administered with n-hexane extract. **(E)** Cross sections of rat’s liver of group V treated with cisplatin drug. The section of the normal liver revealed normal liver parenchyma with rows of normal hepatocytes and adjacent sinusoids. The central vein was also unremarkable. No inflammation, dysplasia, or fibrosis was found.

Hematoxylin-eosin-stained thin liver sections extracted from different rat groups (groups I, II, III, IV, V) were examined for histopathology observations. Gross exams revealed normal liver parenchyma, showing group I rows of benign hepatocytes and adjacent sinusoids. The portal triad and central vein were unremarkable. No areas of fibrosis, hemorrhage, or fatty alterations were found in vehicle control group I. No evidence of inflammation or dysplasia was observed in histopathological liver slices of mice in this group (Figure 6A). However, histopathological findings of positive group II (treated As_2_O_3_) revealed observable cellular changes in the livers of the animals, including lipid-filled hepatocytes (hepatic fatty changes), prominent hepatic sinusoids that are engorged with red blood cells (sinusoidal congestion), central hepatic venous congestion, and areas of severe fibrosis. Fibrosis was observed in perioral areas and diffusely present in most of the other hepatic tissue (Figure 6B).

Gross exams revealed no significant notable differences in the morphology of the experimental rat’s liver compared to the control group after extract treatment at 200 mg/kg body weight. The ethanol and n-hexane extracts of the plant showed recovery effects, which significantly improved normal hepatic textures after induction of arsenic toxicity. It was observed that the normal hepatocytes with the unremarkable portal triad and central vein were restored in *G. latifolia* (ethanol and n-hexane) extracts administered in groups III and IV. However, mild fatty changes and fibrosis were observed in the liver of rats from these groups (Figures 6C, D). The cross-histological evaluation of liver sections belonging to group V (treated with cisplatin standard anticancer drug) revealed the most effective recovery of hepatic injury by restoring normal hepatocytes with adjacent sinusoids with unremarkable central vein, mild fatty changes and no fibrosis (Figure 6E). Inflammation and dysplasia were also not observed in any case.

### 3.8 Immunological biomarker analysis

Liver cancer biomarkers were assessed in all treated groups in comparison with the control and toxic groups and analysis was performed triplicate. The estimations of individual biomarkers were analyzed in mean values and standard deviation. The comparison among different groups was assessed based on *p*-value (*p* < 0.05). The outcomes in different treated versus control and toxic groups are presented in Table 1. The mean levels of COX-2 were significantly increased in toxic group II. The n-hexane extract showed some good activity in lowering the COX-2 levels in group IV. The mean IL-6 level of 31.18 ± 0.7315 increased significantly in toxic group II. The IL-6 normal levels (close to the control group) refurbished in all the treated groups. HSP90 level of 34.27 ± 1.301 increased significantly (<0.05) in toxic group II. The HSP90 normal levels close to the control group were restored in treated groups. The observed mean value of MMP-3 in the control group was 759.4 ± 6.653. Overall significant statistical difference in mean serum MMP-3 level was observed in the treated and the control group as compared to the toxic group II. The mean VEGF level increased significantly in toxic group II. The VEGF normal levels (close to the control group) were restored in treated groups. The restoration of VEGF was however high in the IV (treated with *G. latifolia* n-hexane extract) group. Overall significant statistical (*p* < 0.05) differences in mean vascular endothelial growth factor levels were observed in the treated and the control groups in comparison to the toxic group.

**TABLE 1:** Levels of Immunological markers in control and groups treated with ethanol and n-hexane extracts of *Gardenia latifolia*.

Parameters	Control group I	Positive control group II	EtOH treated group III	n-hexane treated group IV	Cisplatin-treated group V
COX-2 (ng/mL)	2.277 ± 0.3036	27 ± 1.491	12 ± 0.1491	10.6 ± 0.2582	2.79 ± 0.2923
IL-6 (pg/mL)	2.64 ± 0.3748	31.18 ± 0.7315	10.65 ± 0.4673	8.68 ± 0.5116	2.91 ± 0.4771
HSP90 (ng/mL)	10.84 ± 0.4452	34.27 ± 1.301	13.99 ± 0.1663	13.99 ± 0.1663	11.5 ± 0.2708
MMP-3 (ng/mL)	759.4 ± 6.653	1307 ± 73.9	861.7 ± 3.129	854.6 ± 3.921	782 ± 2.16
VEGF (pg/mL)	210.3 ± 7.072	649.9 ± 8.089	308.8 ± 4.492	290.3 ± 6.219	236.6 ± 4.142
*p-value*	0.0001***	0.0001***	0.0001***	0.0001***	0.0001***

(Data are expressed as mean ± S. D and statistical significance are considered as *p* < 0.05*, 0.001**, and *p* < 0.0001***).

### 3.9 Molecular docking

*In silico* docking, the study was performed using PyRx tools Autodock Vina to proceed with molecular interactions and investigate the mechanical interactions of plant extracts with the selected targets. The best metabolite from each library was selected against each target based on the binding affinity. Further interaction analysis was taken place to select the best pose. The binding affinity of the selected metabolites is listed in Table 2.

**TABLE 2:** The top metabolites along with their binding affinity against target proteins.

Target	PDB ID	PubChem ID	Metabolite name	PubChem ID	Docking score
COX-2	5KIR	5280794	Stigmasterol	5280794	−8.3
IL-6	1ALU	5280794	Stigmasterol	5280794	−7.1
VEGF	1FLT	12922960	6-AH-cAMP	12922960	−7.5
MMP-3	1D5J	6100671	Dasycarpidan-1-methanol, acetate (ester)	550,072	−6.8
HSP-90	1YET	6100671	Dasycarpidan-1-methanol, acetate (ester)	550,072	−7.2

**FIGURE 7:**
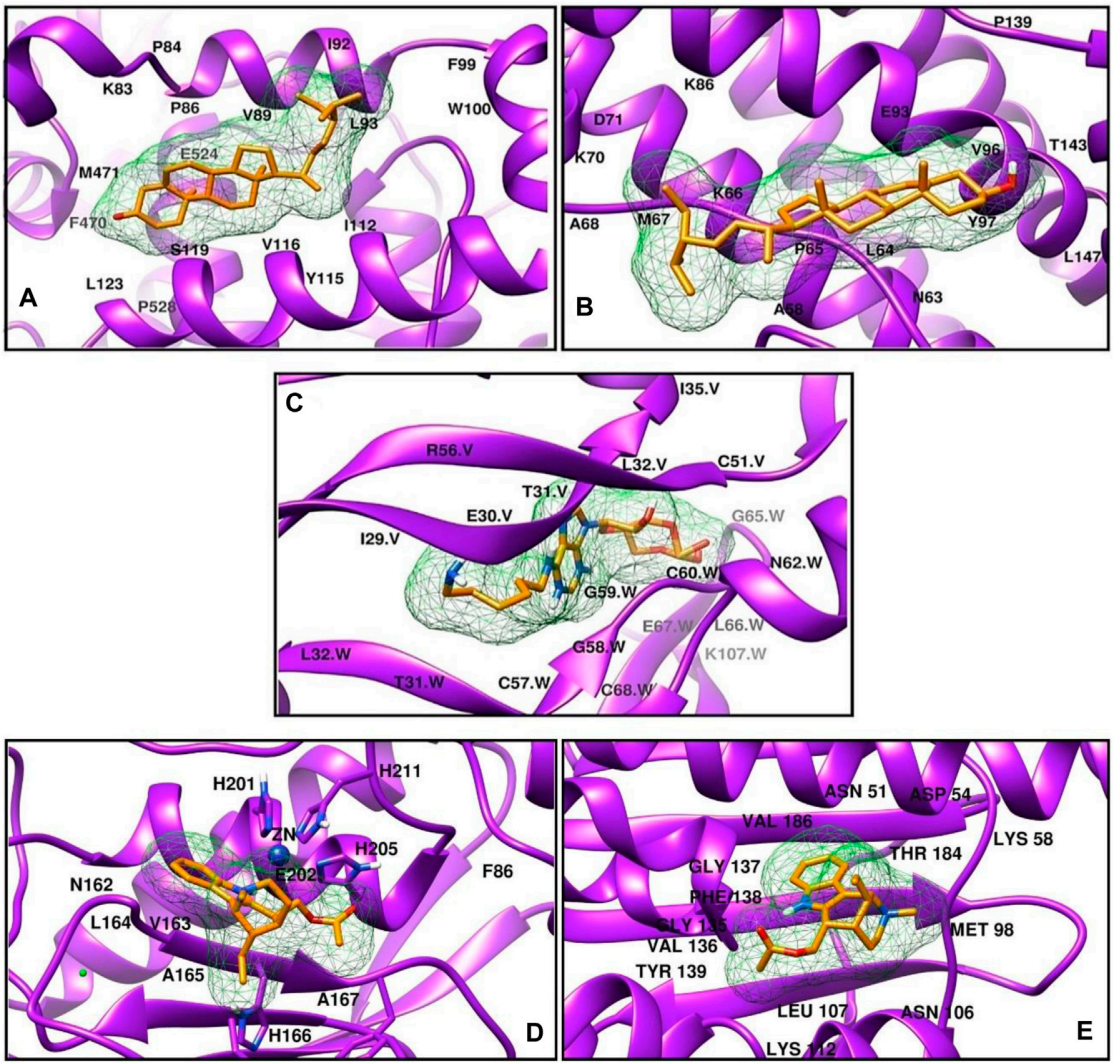
The three-dimensional interaction diagrams of G. *latifolia* extracts **(A)** COX-2-Stigmasterol **(B)** IL-6-Stigmasterol **(C)** VEGF-6-AH-cAMP **(D)** MMP-3- Dasycarpidan-1-methanol, acetate. **(E)** HSP- Dasycarpidan-1-methanol, acetate.

Stigmasterol showed the best binding affinity against COX-2 and IL-6 from the *G. latifolia* plant-extracted compound (Table 2). This metabolite formed mainly hydrophobic interactions with COX-2. The residues Val89, Ile92, Leu93, Trp100, Arg120, and Leu123 of COX-2 were establishing Alkyl and Pi-Alkyl interactions with the metabolite-5280794. In contrast, it was observed to form one hydrogen bond with Asn144, which involved Ala58, Pro65, Ala68, and Lys70 residues of IL-6 in hydrophobic interactions, as shown in (Figures 7A, B). 6-AH-cAMP showed rich interactions with the VEGF protein. The conventional hydrogen bonds were observed between the metabolite and Glu30, Leu32, Asp34, and Ser50 of chain V while residues Asn62 and Asp63 of chain W. Moreover, the Glu30 of chain W and Asp34 of chain V also formed carbon-hydrogen bonds. The hydrophobic interactions were formed between Ile29 and Glu64 of chain W (Figure 9 Cfig9). The metabolite Dasycarpidan-1-methanol, acetate (ester) showed the best binding affinity against the MMP-3 and HSP-90 receptors (Table 2). With MMP-3, it formed hydrogen bonds with Glu202, whereas Leu164 formed Pi donor hydrogen bond and His 211 and Val 163 and his166 formed pi-pi T-shaped, Alkyl and Pi alkyl bonds respectively. In addition, Val398, Leu188, and His401 were involved in Alkyl and Pi-Alkyl interactions. In the case of HSP-90, it formed hydrogen bonds with Lys112 and Phe138, (Figures 7D, E).

**FIGURE 8:**
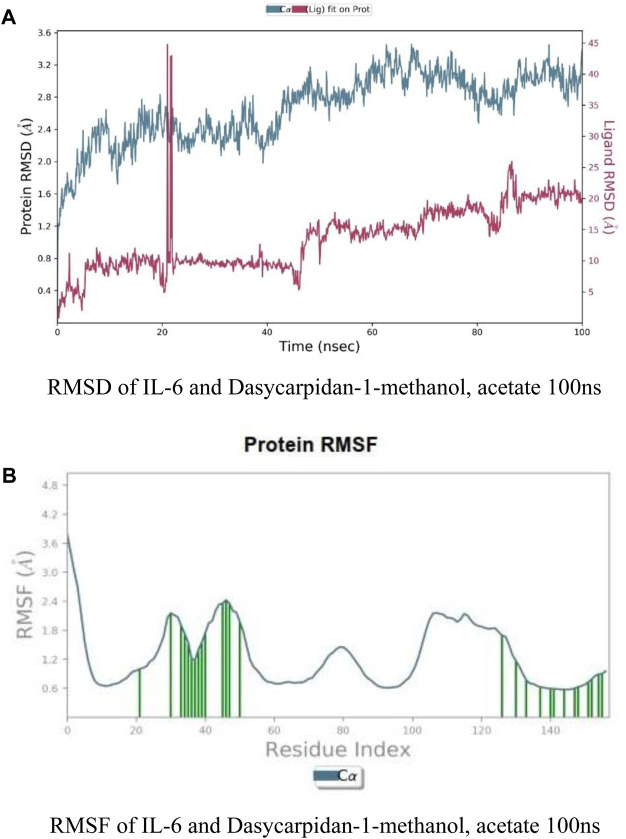
**(A)** RMSF of IL-6 and Dasycarpidan-1-methanol, acetate 100 ns. **(B)** RMSF of IL-6 and Dasycarpidan-1-methanol, acetate 100 ns.

It was identified from literature studies that the target protein 1L-6 possess effective anticancer properties, so it was selected for further docking interactions and similarly selected ligand Dasycarpidan-1-methanol, acetate (ester) was selected due to its drug like properties (as identified from Swiss ADME online server), hence MD simulation was performed by using Gromacs 3.0 simulation software. RMSD of both protein (1L-6) and ligand (Dasycarpidan-1-methanol, acetate (ester)) was represented for the time duration of 100 ns (Figure 8A). There was an instant increase in RMSD of ligand after first 20 ns duration in simulation which representing the gradual change in structure of protein to form the complex. Average RMSD of protein was 2.9 Å while RMSD of ligand was 0.10 Å, which indicated the stability of complex as the difference between RMSD of apo protein and ligand was less than 3 Å. At end, this graph showed the stable dynamic nature of protein ligand complex.

RMSF of protein was plotted for 100 ns which indicated that how much whole protein was flexible in order to fit the ligand molecule to become active in biological system during the simulation course. In this plot (B), residues from 1–20 possessed lower flexible region while residues from 20–60 are more flexiblewith respect to ligand molecule, may be this region possess the binding pocket residues and show more flexibility after binding to ligand. Residues from 100–140 also possessed flexible region while residue 140–156 were higher flexible with respect to ligand.

## 4 Discussion

Developing novel chemotherapeutic agents is crucial to identifying efficient cytotoxic medications with few adverse effects on the surrounding healthy tissues. The hardest cancer to cure is HCC, and it is the most common type of primary liver cancer. The development of several new therapeutic techniques, including plant extract with outstanding anti-proliferative ability, has been facilitated by advancements in the treatment of hepatocarcinogenesis (Mohamed et al., 2022). The effectiveness of plants is well recognized from the interaction of various metabolites present in plants as a whole or in isolated forms. We investigated the anticancer effects of medicinally important plant *G. latifolia* ethanolic and n-hexane extracts against HCC to prove this concept. It was observed from the present study that, the ethanol and n-hexane extracts of *G. latifolia* have antiproliferative and antioxidative properties and may be a useful candidate for discovering new therapeutic options against liver cancer.

According to a study, the bark of this medicinal plant has more phytochemical potency than the leaf and fruit sections, as total phenolics, flavonoids, and tannins were found to be greater in the bark. Methanolic extracts of leaves stems and fruit had IC_50_ values of 145.83, 79.74, and 117.93 μg/mL, respectively, observed from the DPPH scavenging activity. The total antioxidant activity revealed a similar pattern, with bark having the highest antioxidant potential (41.20 mg AAE/g), followed by fruit (31.23 mg AAE/g) and leaves (13.45 mg AAE/g) (Ansari et al., 2019; Alshabi and Shaikh, 2022).

The ethanol extract showed the highest antioxidant activity compared to the n-hexane extract of the plant. Similar results were also presented in a previous study that demonstrated the enormous potential of *G. latifolia* fruits. It was shown that the methanolic fruit extract demonstrated strong antioxidant activity due to the presence of phenolic metabolites (Sundar and Habibur, 2018). Similar observations were reported that *G*. *latifolia* bark hydroalcoholic extract possesses substantial pharmacological activities. Extracts offered significant antioxidant potential regarding their phytochemical content and free radical scavenging ability (Somaida et al., 2020). *Gardenia latifolia* fruits have been used to alleviate fever, colic, and afflictions of the mammary glands. Fruit extracts were used to heal wounds, foot sores, and snake bites. Saponins obtained from bark reduce histamine production, reduce oxidative stress levels, and may be utilized to treat bronchial asthma (Selim et al., 2022). The DPPH assay and MIC determination methods were used to measure membrane-stabilizing and antioxidant activity. The various soluble fractions of *G. latifolia* were tested for their ability to scavenge DPPH radicals, and a dose-response curve of this activity was discovered (Elizondo-Luévano et al., 2022). Our findings showed that higher concentrations of ethanol, and n-hexane extracts were shown to exhibit dose-dependent toxicity in the HepG2 cells. The cytotoxicity and anti-inflammatory potential of *G*. *latifolia* were described, and this plant was the subject of research to find natural phytochemicals. Based on its anti-inflammatory properties, the extract significantly reduced the infected cell’s ability with an IC_50_ value of 10.93 μg/mL and a selectivity index (SI) of 12.88 compared to gold standard antiviral acyclovir (Vindhya and Leelavathi, 2015). Based on a study, it has been shown that the main components that may have cytotoxic effects include phytochemical compounds such terpenoids and flavonoids. *Gardenia latifolia* exhibited potential cytotoxicity, as evidenced by an increase in percentage mortality with increasing concentration. Nevertheless, further research needs to be done to identify and define the particular bioactive principles (Vindhya and Leelavathi, 2014). The anticancer potential of *G. latifolia* against breast cancer was explained in a study that plant leaves were tested for cytotoxicity against MCF-7 cell lines using the MTT assay. The amount of ethanolic extract produced a CTC_50_ value of 170.00 ± 2.0 μg/mL. The study’s outcome supported our cytotoxicity assay observations that *G*. *latifolia* may have cytotoxic potential against cancer cells (Hamza et al., 2021).

Cisplatin is a chemotherapeutic medication that is frequently used to treat liver cancer. Cisplatin stops DNA replication by crosslinking DNA strands. By doing this, cancer cells are unable to multiply, which eventually results in cell death. Cancer cells undergo apoptosis, or programmed cell death, as a result of the harm induced by cisplatin-mediated DNA crosslinking (Siddik and Tchounwou, 2014). According to immunohistochemical staining in rat livers, using plant extract as an antioxidative metabolite was supported by research findings which elaborated that ginger extract inhibited the rise in the number of cells positive for COX-2. By preventing cell development and triggering apoptosis, ginger extract has a significant chemo-preventative effect on liver cancer. Ginger protects against cancer in rat liver by minimizing oxidative and inflammatory damage (Hamza et al., 2021).

Plant-derived anticancer drugs induce apoptosis in tumor cells through specialized mechanisms. Cancer cells exploit metabolic changes to escape apoptosis caused by oxidative damage, enabling rapid growth (Reddy and Reddanna, 2009). Our findings observed that *G. latifolia* extract decreased scavenging activities of H_2_O_2_, nitric oxide, superoxide, and DPPH, reducing oxidative stress and enhancing the antiproliferative effectiveness compared to untreated HepG2 cells and normal BHK cells. A molecule of 1-acetyl 4, 5-di-O-caffeoylquinic acid was observed, which was obtained from the phytochemical examination of *G. latifolia*. With significant tumor selectivity, the identified substance was the most cytotoxic against the colon cancer cell line (IC_50_ 1.9 μg/mL). Findings also demonstrated considerable *in silico* topoisomerase II inhibition, G2/M cell cycle arrest, and apoptotic abilities due to the antiproliferative effects of this plant (Abass et al., 2018).

Liver functions show biochemical and hematological marker alterations due to the destructive effects of As_2_O_3_ on tissues. These changes indicate organ damage caused by arsenic toxicity (Amin et al., 2012). DNA damage can occur when arsenic chemicals interact directly with DNA. In addition to causing genomic instability and the buildup of mutations, which are characteristics of the development of cancer, this damage can obstruct normal DNA replication and repair activities. By inhibiting antioxidant enzymes and interfering with mitochondrial activity, among other methods, arsenic can produce reactive oxygen species (ROS) (Ray et al., 2014).

A study discussed the effect of *Alpinia officinarum* rhizome extract in cisplatin-treated rats with HCC. Results revealed that DENA elevated hepatic indices such as ALT and AST with declining serum total protein and induced oxidative stress with depressed SOD and CAT activities. Hepatocarcinogenesis was also detected by histopathological changes in liver sections, similarly to those observed in our findings. Treatment with cisplatin partially restored altered hepatic functions and oxidative stress markers. The treatment with AORE + CP enhanced hepatic function and oxidative stress markers. This effect was more potent than the treatment with CP alone (Elizondo-Luévano et al., 2022). Another study supported histopathological observations of our work and explained that gene expression analyses in Mahua extract (ME) treated HepG2 cells revealed a considerable downregulation of the proteins AKT1/2/3, p-AKT, and COX-2, as well as an upregulation of active caspase-3. The mice were pretreated with Mahua extract for *in vivo* tests. Histological examinations of the cirrhotic mouse liver revealed CCl_4_-induced centrilobular necrosis and fatty vacuoles. This study demonstrated ME-mediated antioxidant activity and hepatoprotective effects; therefore, it could be used for treating hepatic disorders, including liver cancer, especially in combination with chemotherapeutics (Abass et al., 2018). Anticancer activity against HCC was observed in the Mongolian medicinal plant *S. involucrata,* which also possesses anti-inflammatory and analgesic properties. *In vitro* tests were performed on HCC cells in response to *Saussurea involucrata* extract (SIE) to assess their cytotoxicity AO/EB staining for apoptotic cells. The normal signs of apoptotic cell death were seen in cells exposed to SIE (Pi et al., 2002).

Liver cancer biomarkers were assessed in all groups through COX-2, MMP-3, HSP90, VEGF (vascular endothelial growth factor), and Interleukin-6 (IL-6). Elevated levels of cancer biomarkers were observed in the toxic group that refurbished in *G*. *latifolia* ethanol and n-hexane extracts. A previous study showed the efficient effects of ethanol and n-hexane extracts of *G*. *latifolia* in combating carcinoma (Byambaragchaa et al., 2014). The biomarkers that were described had the greatest diagnostic value in histological analyses and may be indicative of the immunological status of various illnesses. Preliminary analysis of these markers before the initiation of therapy may be utilized to develop a treatment plan that includes immunomodulatory medications, probiotics, inhibitors of pro-inflammatory cytokines, and checkpoint inhibitors (Ray et al., 2014).

From the obtained results of the antioxidant assay of *G*. *latifolia* plant extracts, it is understandable that ethanolic extracts revealed higher antioxidant activity and might be attributed to the presence of a significantly high number of antioxidant substances as analyzed during the phytochemical and antioxidant assays. It was found that the therapeutic undertaking of distinct metabolites of *G*. *latifolia* is well correlated with the phenolic, flavonoid, and tannin contents of the plant, which illustrate the species as a powerful supply of antioxidants and concurrently encourage the scientific world to look at the medicinal properties of this medicinal plant as a therapeutically energetic, botanical drug resource. *Gardenia latifolia* has potential cytotoxicity against liver cancer cells. However, further investigations are needed to isolate and characterize the specific bioactive principles.

## 5 Conclusion

The medicinal values of *G. latifolia* extracts against liver cancer were estimated in this study. The study revealed the presence and high content of the important phytochemicals. The plant extracts resulted in high antioxidant activities. The ethanolic extract showed higher antiproliferative, and antioxidative activities in comparison with n-hexane extract against cancer cells. Furthermore, *in vivo,* studies revealed the efficacy of the plant in lowering the damage caused by arsenic toxicity. The effect of the plant extracts was further confirmed by the estimation of cancer markers. Additionally, *in silico* molecular docking revealed some good binding affinities of the identified plant metabolites with cancer marker targets. Overall, the plant ethanolic and hexane extracts resulted in some promising outcomes in lowering the effects of liver cancer. However, further studies on cellular and metabolic levels will be useful in the early and rapid estimation of the plant potential against various types of cancer.

## Data Availability

The original contributions presented in the study are included in the article/Supplementary Material, further inquiries can be directed to the corresponding authors.

## Abbreviations

HCC, Hepatocellular carcinoma; GL, Gadenia latifolia; GC-MS, Gas chromatography-mass spectrometry; As2O_3_, Arsenic trioxide; ECM, Extracellular matrix; WHO, World health organization; PAMPs, Pathogen-associated molecular pathogens; ROS, Reactive oxygen species; IL-6, Interleukin-6; IL-1β, Interleukin-1 beta; TNF-α, Tumor necrosis factor-alpha; COX-2, Cyclooxygenase-2; NF-кβ, Nuclear factor kappa-light-chain-enhancer of activated B cells; DPPH (2, 2-diphenyl-1-picrylhydrazyl); AlCl3, Aluminum chloride; NaNO2, Sodium nitrite; NaOH, Sodium hydroxide; PBS, Phosphate buffer saline; NBT, Nitroblue tetrazolium; NADH, Nicotinamide adenine dinucleotide; FBS, Fetal Bovine serum; DMEM-HG, Dulbecco’s modified Eagle medium; HEPES, 2-[4-(2-hydroxyethyl)piperazin-1-yl]ethanesulfonic acid; EDTA, Ethylenediamine tetraacetic acid; DTNB, 5, 5′-Dithiobis(2-nitrobenzoic acid); TCA, Trichloroacetic acid; ELISA, Enzyme linked immunosorbent assay.
