# Supplementary material for: Phytochemical profiling and anticancer potential of gardenia latifolia extracts against arsenic trioxide induced liver fibrosis in rat model
Source: Front Pharmacol. 2024 Aug 30;15:1389024. doi: 10.3389/fphar.2024.1389024 (PMC11395838; doi:10.3389/fphar.2024.1389024)
Supplement: Supplementary file 1 [file DataSheet1.docx]

**Supplementary Table 1:** List of identified compounds in the ethanol extract of G. latifolia using GC-MS analysis.

| Peaks No. | Compound Name | Mol. Wt.  m/z | Mol. Formula | Retention Time (RT) | Class of Compounds |
| --- | --- | --- | --- | --- | --- |
| 1 | Benzene, 1-ethyl-3-methyl- | 120 | C_9_H_12_ | 3.57 | Aromatic hydrocarbon |
| 2. | Decane | 142 | C_10_H_22_ | 4.02 | Aliphatic hydrocarbon |
| 3 | 12,15-Octadecadiynoic acid, methyl Ester | 290 | C_19_H_30_O_2_ | 4.47 | Fatty acid ester |
| 4 | Hexyl octyl ether | 274 | C_10_H_17_Cl_3_O_2_ | 5.00 | Ether |
| 5 | Undecane | 156 | C_11_H_24_ | 5.69 | Aliphatic hydrocarbon |
| 6 | Heptadecane, 9-hexyl- | 324 | C_23_H_48_ | 7.38 | Alkane hydrocarbon |
| 7 | Benzene, 1,3-bis(1,1-dimethylethyl)- | 190 | C_14_H_22_ | 8.24 | Aromatic hydrocarbon |
| 8 | Octadecane, 1-chloro- | 242 | C_16_H_34_O | 8.66 | Alkyl halide |
| 9 | Octadecanoic acid, 4-hydroxy-, methyl ester | 314 | C_19_H_38_O_3_ | 9.09 | Fatty acid ester |
| 10 | Picrotoxinin | 292 | C_15_H_16_O_6_ | 9.72 | Furopyrans |
| 11 | cis-1-Chloro-9-octadecene | 286 | C_18_H_35_Cl | 9.89 | Alkenyl halide |
| 12 | 14-Octadecenal | 266 | C_18_H_34_O | 10.62 | Aldehyde |
| 13 | 2-Hexadecanol | 242 | C_16_H_34_O | 12.02 | Secondary alcohol |
| 14 | Neopentyl glycol, dibenzoate | 312 | C_19_H_20_O_4_ | 12.41 | Benzoate esters |
| 15 | Ingol 12-acetate | 408 | C_22_H_32_O_7_ | 12.72 | Allylbenzene |
| 16 | Hexadecane, 1,1-bis(dodecyloxy)- | 594 | C_40_H_82_O_2_ | 13.57 | Ether |
| 17 | Tetradecanoic acid, ethyl ester | 256 | C_16_H_32_O_2_ | 16.97 | Fatty acid esters |
| 18 | 3,7,11,15-Tetramethyl-2-hexadecen-1-o1 | 296 | C_20_H_40_O | 17.42 | Diterpene |
| 19 | 11,13-Dimethyl-12-tetradecen-1-ol Acetate | 282 | C_18_H_34_O_2_ | 17.87 | Fatty acid |
| 20 | Hexadecanoic acid, ethyl ester | 284 | C_18_H_36_O_2_ | 18.89 | Long-chain fatty acid ester |
| 21 | 9,12-Octadecadienoic acid, ethyl ester | 308 | C_20_H_36_O_2_ | 20.11 | Fatty acid ester |
| 22 | Linoleic acid ethyl ester | 308 | C_20_H_36_O_2_ | 21.97 | Long-chain fatty acid ester |
| 23 | Dasycarpidan-1-methanol, acetate (ester) | 326 | C_20_H_26_N_2_O_2_ | 22.54 | Alkaloidal compound |
| 24 | 1-Monolinoleoylglycerol trimethylsilyl Ether | 498 | C_27_H_54_O_4_Si_2_ | 22.92 | Alkaloidal compound |
| 25 | E,E,Z-1,3,12-Nonadecatriene-5,14-diol | 294 | C_19_H_34_O_2_ | 24.49 | Alkenyl diols |
| 25 | Oxalic acid, octadecyl propyl ester | 384 | C_23_H_44_O_4_ | 24.94 | Dicarboxylic acid |
| 26 | Stigmasterol | 412 | C_29_H_48_O | 25.69 | Stigmastanes |

**Supplementary Table 2:** List of identified compounds in the n-hexane extract of G. latifolia using GC-MS analysis.

| Peak No. | Compound Name | Mol. formula | Mol. wt.  m/z | Retention Time | Class of compound |
| --- | --- | --- | --- | --- | --- |
|  | Cyclopentasiloxane, decamethyl- | C_10_H_30_O_5_Si_5_ | 370 | 6.60 | Organosilicon compound |
|  | Dodecane | C_12_H_26_ | 170 | 7.29 | Aliphatic hydrocarbon |
|  | Benzene, 1,3-bis(1,1-dimethylethyl)- | C_14_H_22_ | 190 | 8.21 | Alkylbenzene |
|  | Tridecane | C_13_H_28_ | 184 | 8.94 | Aliphatic hydrocarbon |
|  | 6-AH-cAMP | C_16_H_24_N_6_O_6_P | 427 | 9.41 | cyclic AMP |
|  | 1,2-15,16-Diepoxyhexadecane | C_16_H_30_O_2_ | 254 | 9.78 | Epoxides |
|  | 2-Myristynoyl pantetheine | C_25_H_44_N_2_O_5_S | 484 | 10.15 | Amide |
|  | Tetradecane | C_14_H_30_ | 198 | 10.53 | Aliphatic hydrocarbon |
|  | Hexadecane, 2,6,10,14-tetramethyl- | C_20_H_42_ | 282 | 11.49 | Substituted hydrocarbon |
|  | Phenol, 2,4-bis(1,1-dimethylethyl)- | C_14_H_22_O | 206 | 12.31 | Phenolic compound |
|  | Dodecanoic acid, ethyl ester | C_14_H_28_O_2_ | 228 | 13.45 | Fatty acid esters |
|  | Dodecanoic acid, 1-methylethyl ester | C_15_H_30_O_2_ | 242 | 13.96 | Fatty acid esters |
|  | Limonen-6-ol, pivalate | C_15_H_24_O_2_ | 236 | 14.22 | Sesquiterpene |
|  | 1-Hexadecanol, 2-methyl- | C_17_H_36_O | 256 | 15.24 | Alcohol |
|  | 9-Octadecenoic acid, (2-phenyl-1,3-dioxolan-4-yl)methyl ester, cis- | C_28_H_44_O_4_ | 444 | 15.83 | Ester |
|  | 7-Methyl-Z-tetradecen-1-ol acetate | C_17_H_32_O_2_ | 268 | 16.16 | Carboxylic ester |
|  | Tetradecanoic acid, ethyl ester | C_16_H_32_O_2_ | 256 | 16.87 | Fatty acid ester |
|  | Isopropyl Myristate | C_17_H_34_O_2_ | 270 | 17.26 | Fatty acid ester |
|  | Hexadecanoic acid, ethyl ester | C_18_H_36_O_2_ | 284 | 17.70 | Fatty acid ester |
|  | Pentadecanoic acid, 14-methyl-, methyl Ester | C_17_H_34_O_2_ | 270 | 18.27 | Fatty acid ester |
|  | Linoleic acid ethyl ester | C_20_H_36_O_2_ | 308 | 20.21 | Long-chain fatty acid |
|  | 9,12,15-Octadecatrienoic acid, 2,3-dihydroxypropyl ester, (Z,Z,Z)- | C_21_H_36_O_4_ | 352 | 21.29 | Fatty acid ester |
|  | 9-(2',2' Dimethylpropanoilhydrazono)-3,6-dichloro-2,7-bis-[2-(diethylamino)-ethoxy] fluorine | C_30_H_42_Cl_2_N_4_O_3_ | 576 | 22.33 | Fluorinated heterocycles |
|  | 17-Pentatriacontene | C_35_H_70_ | 490 | 23.02 | Olefine hydrocarbon |
|  | Ethyl tetracosanoate | C_26_H_52_O_2_ | 396 | 23.51 | long-chain fatty acyl coas |
|  | 1-Heptacosanol | C_27_H_56_O | 396 | 24.00 | Fatty alcohols |
|  | Oxalic acid, octadecyl propyl ester | C_23_H_44_O_4_ | 384 | 25.00 | Ester |
|  | α-Sitosterol | C_29_H_50_O | 414 | 25.97 | Phytosterols |
|  | 7aH Cyclopenta[a]cyclopropa[f]cyclondecene-2,4,7,7a,10,11-hexol, 1,1a,2,3,4,4a,5,6,7,10,11,11a-dodecahy dro-1,1,3,6,9-pentamethyl-, 2,4,7,10,11-pentaacetate | C_30_H_44_O_11_ | 580 | 26.65 | Cyclic alkene |
|  | 3,7,11,15-Tetramethyl-2-hexadecen-1-o1 | C_20_H_40_O | 296 | 27.17 | Acyclic diterpenoids |
|  | Glycine, N-[(3à,5á,7à,12à)-24-oxo-3,7,12-tris[(trimethylsilyl)oxy] cholan-24-yl]-, methyl ester | C_36_H_69_NO_6_Si_3_ | 695 | 27.66 | Steroidal compound |
|  | 9,10,12,13-Tetrabromooctadecanoic acid | C_18_H_32_Br_4_O_2_ | 596 | 28.86 | Long-chain fatty acid |
